# Dramatic Dose Reduction in Three-Dimensional Rotational Angiography After Implementation of a Simple Dose Reduction Protocol

**DOI:** 10.1007/s00246-018-1943-3

**Published:** 2018-08-03

**Authors:** Savine C. S. Minderhoud, Femke van der Stelt, Mirella M. C. Molenschot, Michel S. Koster, Gregor J. Krings, Johannes M. P. J. Breur

**Affiliations:** 1 Department of Pediatric Cardiology, Wilhelmina Children’s Hospital, University Medical Center, Lundlaan 6, 3584 EA Utrecht, The Netherlands; 2 Radiation Protection and Consultancy, NRG-Consultancy and Services, Westerduinweg 3, 1755 LE Petten, The Netherlands

**Keywords:** Catheterization, Rotational angiography, Radiation dose reduction, Effective dose

## Abstract

Previously, median effective dose (ED) of 1.6 mSv per three-dimensional rotational angiography (3DRA) has been reported. This study evaluated ED and image quality in 3DRA after implementation of a simple dose reduction protocol in pediatric catheterizations. Simple conversion factors between 3DRA ED and readily available parameters at the cathlab were determined. The dose reduction protocol consisted of frame reduction (60–30 frames/s (f/s)), active collimation of the X-ray beam, usage of a readily available low dosage program, and a pre-3DRA run check. EDs were calculated with Monte Carlo PCXMC 2.0. Three observers blindly assessed 3DRA image quality of the dose reduction and normal-dose cohort. Between October 2014 and October 2015, 84 patients (median age 4.3 years) underwent 100 3DRAs with a median ED of 0.54 mSv (0.12–2.2) using the dose reduction protocol. Median ED in the normal-dose cohort (17 3DRAs) was 1.6 mSv (1.2–4.9). Image quality in the dose reduction cohort remained excellent. Correlations between ED and dose area product (DAP) and ED and skin dose were found with a *ρ* of 0.82 and 0.83, respectively. ED exposure of the entire catheterization was reduced to 2.64 mSv. Introduction of a simple protocol led to 66% dose reduction in 3DRA and 79% in the entire catheterization. 3DRA image quality in this group remained excellent. In 3DRA ED correlates well with DAP and skin dose, parameters readily available at the cathlab.

**Electronic supplementary material:**

The online version of this article (10.1007/s00246-018-1943-3) contains supplementary material, which is available to authorized users.

## Introduction

In pediatric cardiology imaging is essential for diagnostic and interventional purposes. For this reason, patients with congenital heart disease regularly and increasingly receive radiation over the years [1]. Radiation exposure during childhood is more harmful than during adulthood. Reasons for this are the longer life span children have and the more harmful effects radiation has on developing tissue. With increased radiation exposure, children’s lifetime cancer risks will increase [2–5]. Catheterizations contribute to the majority of radiation burden in patients with congenital heart disease [6].

Quite recently, three-dimensional rotational angiography (3DRA) has been added to the spectrum of image modalities. 3DRA is used for diagnostic and interventional reasons. 3DRA provides a real-time roadmap for anatomy-guided procedures and improves faster and simplified interventions with enhanced patients’ safety [7]. Conversion factors enabling simple estimation of effective dose (ED) for standard procedures such as 3DRA acquisition have not yet been published [8]. Many studies report dose area product (DAP), a value directly provided by the imaging system [9, 10], but this value does not quantify the radiation’s effect on patients. The effective radiation dose (ED) is the best indicator to assess the stochastic effects of radiation [3]. Furthermore, ED enables comparison between the effects of 3DRA and effects of other imaging modalities [3].

Many studies directly estimate ED from DAP [10–12]. However, the relation between DAP and ED for 3DRA has never been strongly confirmed [13]. A strong correlation will help to produce a simple formula to estimate the ED, which is more practical for daily use than the complex ED calculations.

In 2014, Peters et al. have reported a median ED of 1.6 mSv per 3DRA in only 17 pediatric patients [14]. To limit the radiation burden, the ED should be reduced to a minimum with preservation of image quality. The ED might decrease with a few simple changes in the 3DRA protocol [2, 13, 14].

Therefore, the aim of the present study is (1) to calculate the EDs after implementation of a simple dose reduction protocol in a larger group of patients and compare the results with Peters et al., (2) to evaluate imaging quality of this protocol, and (3) to further explore the correlation between DAP and ED [14].

## Materials and Methods

### Study Population

Patients were eligible for inclusion if they were 0–18 years of age and had undergone a cardiac catheterization procedure with 3DRA acquisition at the Wilhelmina Children’s Hospital between October 2014 and October 2015. The institutional review board approved this study and no informed consent was required. Retrospective analysis of medical records and catheterization data was performed. Parameters collected include age, weight, height, body surface area (BSA), cardiac diagnosis, and type of intervention (if applicable). Patients were grouped according to their initial diagnosis. Patient characteristics of this low-dose cohort 3DRA were compared with a patient group previously reported, undergoing a normal-dose 3DRA [14]. Reasons for exclusion from ED calculation were incomplete rotation, wrong positioning of the patient, and insufficient contrast. As contrast absorbs radiation, insufficient contrast leads to less radiation exposure. 3DRAs made with a central venous catheter or because of a non-cardiac diagnosis were excluded from image quality assessment.

### 3DRA Image Acquisition

3DRAs were obtained using the Siemens Artis Zee biplane (Siemens, Forchheim, Germany) and reconstructions were transferred to the Leonardo workstation for post-processing with Syngo DynaCT Cardiac software. All procedures were performed under general anesthesia. Rapid atrial or ventricular pacing was performed in 88 of the 100 3DRAs. Pacing frequency was increased from 180/min upwards until a reduction of 50% of the systolic blood pressure was achieved. Contrast medium was administrated to the cardiac compartment prior to the region of interest meaning the right ventricle for pulmonary imaging and the left ventricle for aortic imaging. Contrast was diluted up to 60% with saline. Contrast was injected from 2 mL/s in 3 kg neonates up to 16 mL/s in 50 kg adolescents in case of a single injection site before start of 3DRA for 5 s. When multiple injection sites were necessary, additional manual injections with diluted contrast were performed.

### Dose Reduction

Compared to the study of Peters et al., the number of frames per second was reduced from 60 to 30 f/s [6]. In addition, patients were scanned with a tube voltage corresponding to a low-dose program (Table S1). All patients were scanned according to a protocol of one weight class lower than the patient’s weight. Furthermore, a thick copper filter was used to filter out low-energy photons that can cause harm and do not contribute to the image quality. Collimation was applied with a diaphragm to protect irrelevant tissue from radiation and to prevent scattering rays from causing background haze. Before the actual run, tube current was checked to be below 100 mA. If not, image plane was checked for metal artifacts and the tube current was automatically adjusted accordingly.

### Calculation of ED

All data required for calculation were extracted from Artis Zee biplane and converted to Microsoft Excel (Microsoft, USA) with CareAnalytics (Siemens, Erlangen, Germany). Parameters describing the geometry of the X-ray tube, the radiation quality (tube current, filter material and thickness, and anode angle), and the patient (age, height, and weight) were imported in Monte Carlo program PCXMC 2.0 (STUK, Finland) from Microsoft Excel to calculate ED. The outcomes of the Monte Carlo stimulations are according to International Commission on Radiological Protection 103 (ICRP103) organ weighing factors [15, 16].

### Image Quality Assessment

3DRA images were extracted from the Leonardo workstation and patient characteristics and cohort information were removed from the files. One experienced pediatric cardiologist and two junior researchers blindly assessed image quality independently. Pre-defined anatomical structures, necessary for diagnosis or intervention, were separately scored on a three-point scale (good = 3, moderate = 2, and poor = 1).

### Statistical Analysis

Continuous values were expressed as median with a range, and categorical values as a number and percentage of the total. Differences between baseline characteristics were tested for significance using T-test or 2-tailed Mann Whitney test, for normally distributed and skewed continuous values, respectively. Significant differences for gender, diagnosis, and image quality were tested with a Chi-square test. *P* < 0.05 was considered to be significant. A Kruskal–Wallis test was used to compare age, weight, and ED per initial diagnosis. Spearman’s correlation testing and linear regression were performed to evaluate the association of ED with patient’s age, height, weight, BSA, skin dose, DAP, and tube current in case of non-normally distributed variables. A Fleiss kappa was calculated to test interobserver agreement of the image quality. All statistical calculations were performed using Microsoft Excel 14.6.1.

## Results

### Exposure Data

Table 1 summarizes characteristics at baseline of the patients undergoing low-dose and normal-dose 3DRAs. For one patient, the height was estimated, because it was not measured during hospital admission. Analysis of baseline parameters between the two groups did not show statistically significant differences. In the low-dose group, 100 runs were performed in 84 patients. Ten patients underwent pre- and post-intervention 3DRA, and four patients had a second catheterization with a 3DRA. Furthermore, two patients had an additional 3DRA for evaluation of a second intervention and further evaluation of a possible vascular ring with esophageal contrast, respectively.

**Table 1:** Baseline characteristics

	Low dose	Normal dose [14]
Demographic patient data		
Number of patients	84	14
Male, *n* (%)	39 (46)	7 (50)
Age (years)	4.29 (0.0–18.8)	3.79 (0–16.6)
Height (cm)	103.0 (50–176)	101.5 (50–184)
Weight (kg)	16.15 (2.4–89)	14.5 (3.4–57.5)
BSA (m^2^)	0.69 (0.19–2.10)	0.65 (0.23–1.68)
Patient diagnosis		
PA + VSD/TOF, *n* (%)	23 (27.4)	4 (28.6)
Aortic pathology, *n* (%)	20 (23.8)	3 (21.4)
Univentricular heart, *n* (%)	19 (22.6)	2 (14.3)
TGA, *n* (%)	6 (7.1)	2 (14.3)
Genetic syndrome, *n* (%)	8 (9.5)	0 (0)
Others, *n* (%)	8 (9.5)	3 (21.4)
Procedural data		
Number of 3DRAs	100	17
Interventional procedures, *n* (%)	75 (75)	12 (71)

Continuous variables are summarized as median and range, and categorical variables are reported as number of cases (*n*) and percentage

*BSA* body surface area, *PA* pulmonary atresia, *TGA* transposition of the great arteries, *TOF* tetralogy of fallot, *VSD* ventricular septal defect

### Dose Reduction

All 3DRAs were made with 30 f/s. In 96 of the 100 3DRA, weight protocols of one weight class lower were used. Table 2 shows the radiation exposure parameters after the dose reduction.

**Table 2:** Technical characteristics

	Low dose *N* = 100	Normal dose [14] *N* = 17	*P* value
Exposure parameters			
Tube voltage 3DRA (kV)	70 (60–96)	90 (90–90)	< 0.001
Tube current 3DRA (mA)	228 (53–395)	69 (26–363)	< 0.001
Exposure time 3DRA (ms)	465.5 (333.2–1330)	843.2 (452.2–868)	< 0.001
Skin dose 3DRA (mGy)	9 (1.7–83)	20,86 (10,33–90,69)	< 0.001
DAP 3DRA (mGy·cm^2^)	1279 (150–16,987)	3128 (1231–17,273)	< 0.001
Procedural time (min)	153 (30–360)	165 (60–540)	0.360
Total fluoroscopy time (min)	28 (0.4–121)	22 (6.4–81.4)	0.497
Effective dose			
3DRA (mSv)	0.54 (0.12–2.23)	1.62 (0.70–4.94)	< 0.001
Fluoroscopy (mSv)	1.53 (0.00–25.40)	4.4 (0.2–15.8)	0.002
Angiography (mSv)	0.45 (0.00–19.33)	3.6 (0–79.1)	0.009
Total catheterization (mSv)	2.64 (0.27–28.13)	12.4 (2–99.9)	< 0.001
Interventional group	3.65 (0.49–28.13)		
Diagnostic group	0.86 (0.27–10.09)		

Values represent median and range

### Effective Dose

**Fig. 1:**
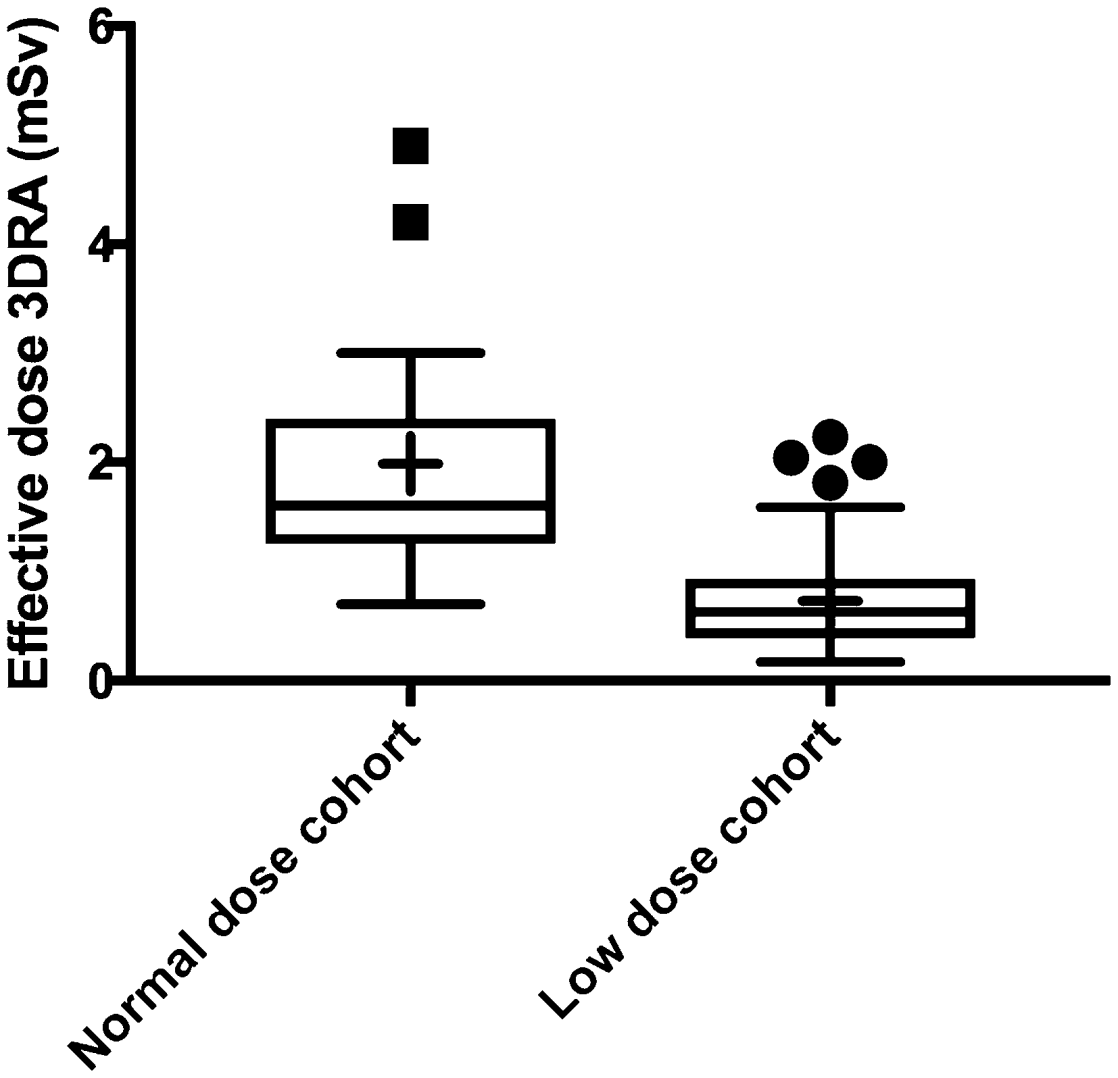
Distribution of 3DRA EDs; + indicates mean value. Normal dose: *n* = 17; low dose: *n* = 100

**Fig. 2:**
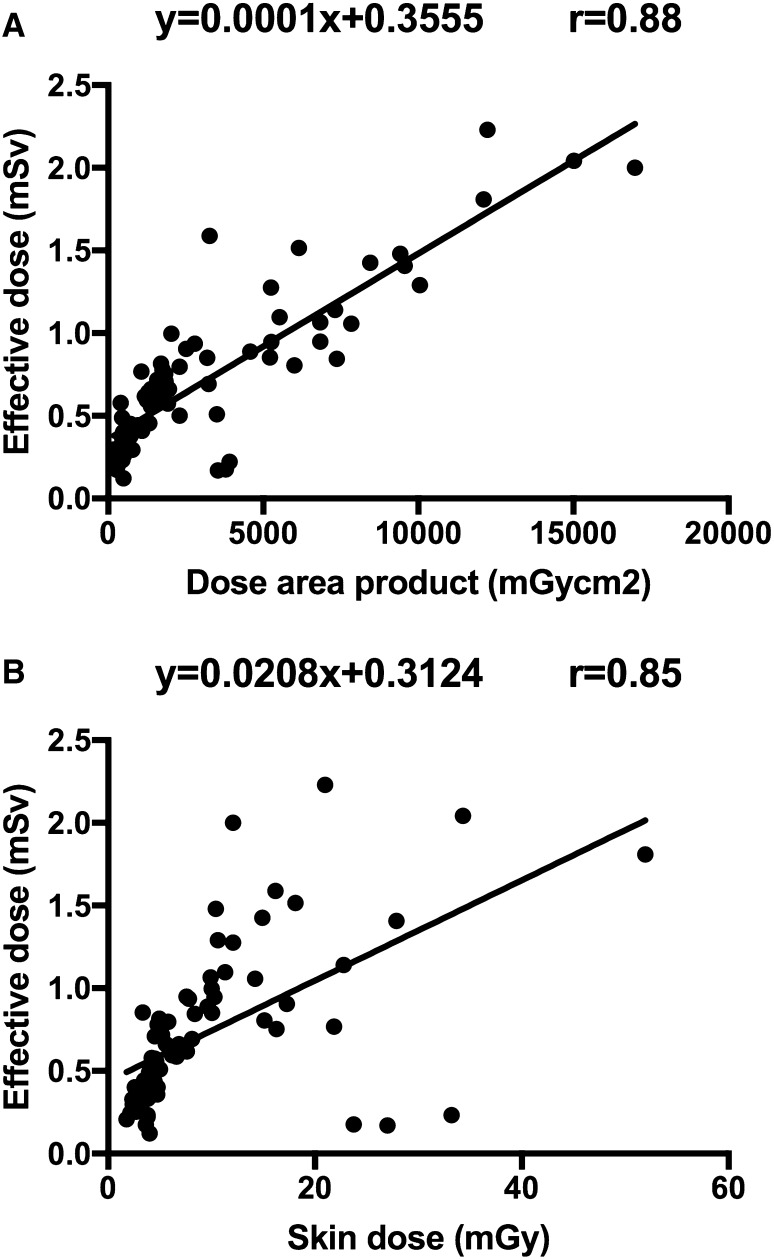
**a** Scatterplot of DAP and ED for patients that underwent a 3DRA in the low-dose cohort—the straight lines indicate the least squared fit for the low-dose cohort. **b** Scatterplot of skin dose and ED for patients that underwent a 3DRA in the low-dose cohort—the straight lines indicate the least squared fit for the low-dose cohort

After applying the dose-reducing protocol, a mean and median ED per 3DRA of 0.67 mSv (± 0.44 SD) and 0.54 mSv (range 0.12–2.2), respectively, were calculated compared to a mean and median ED per 3DRA of 2.0 (± 1.1 SD) and 1.6 mSv (1.2–4.9), respectively, in the normal-dose group (Fig. 1). The reduction in ED achieved with the dose reduction protocol was highly significant (*P* < 0.001, 95% CI 0.82–1.32). Differences in age, weight, and 3DRA ED between diagnostic groups were not significant. Only 2 of the 100 3DRAs had EDs higher than 2.0 mSv. These two patients were aged 15 and 16 years, much higher than median age of 4.3 years. Furthermore, the total procedural ED was reduced from a median total ED 12.4 mSv in the normal-dose group to 2.64 mSv in the low-dose group (*P* < 0.001, 95% CI 2.18–11.28) (Table 2). In patients with a diagnostic catheterization, a median total ED of 0.86 mSv was found. Also, fluoroscopy and angiography ED were reduced, while fluoroscopy and procedure time did not differ between the two groups. DAP and skin dose correlate very well with ED (Fig. 2). Spearman’s correlations coefficients between DAP and ED and skin dose and ED of the 100 low-dose 3DRAs were *ρ* 0.82 and 0.83, respectively. The correlations between DAP and ED and skin dose and ED were the strongest in patients aged 1–4.99 years, *ρ* 0.92 and 0.93, respectively. A multiple linear regression equation was calculated to predict ED based on DAP and weight. A significant regression equation

$${\text{ED}}=0.44 - 0.008 \times {\text{weight}}\left( {{\text{kg}}} \right)+0.000158 \times {\text{DAP}}\left( {{\text{mGy}}{\cdot}{{\text{cm}}^{\text{2}}}} \right)$$

was found (*F*(2,97) = 195.435, *P* < 0.000), with a *R*^2^ of 0.80.

### Image Quality

**Fig. 3:**
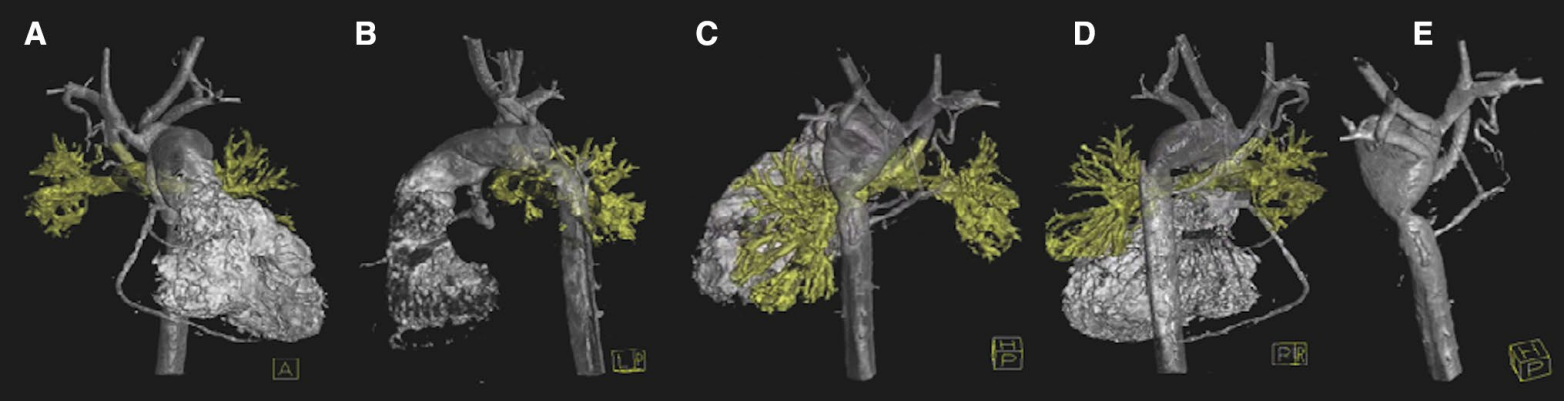
3DRA with an ED of 0.12 mSv—acquired in a 3-month-old female patient with hypoplastic left heart syndrome and aortic coarctation. The overall image quality was sufficient. **a** Anterior view; **b** lateral view; **c–e** posterior views, clearly showing the aortic coarctation

Blind assessment of image quality was possible in 93 of the 112 3DRAs, and those were included in our analysis. The median score in both cohorts was 3; 96% of the items had a median score of good. One anatomical structure in one patient was scored poor (1), and thus overall all images had sufficient quality for clinical decision-making. Comparing the two groups, there was a significantly better image quality in the low-dose cohort. Among the three different reviewers, fair agreement was seen, with a Fleiss kappa value of 0.34. 351 of the 390 (90%) scored items received the same score from all three reviewers. Images of a 3-month-old patient with an aortic coarctation and 3DRA ED of 0.12 mSv are shown in Fig. 3.

## Discussion

With this study, we show that standardized use of traditional 2D angiography dose-lowering techniques leads to a 66% dose reduction in 3DRA with preserved (excellent) image quality.

Furthermore, a significant decrease in total procedural radiation dose was observed. Finally, the strong correlations between DAP and ED and skin dose and ED were confirmed and a simple formula to estimate 3DRA ED was proposed.

### Implications

Annual average EDs are 3 mSv per person of which 80% is due to natural sources such as cosmic radiation. 0.6 mSv of annual average dose results from artificial sources such as medical exposure, atmospheric nuclear testing, and occupational exposure of which medical exposure accounts for almost 100% of the artificial ED [17]. Average annual exposure in pediatric cardiology population varies greatly. Patients with complex congenital heart disease such as a univentricular heart can have an average annual effective dose of 20 mSv solely because of ionizing radiation-producing medical examinations [4]. As a result in patients with complex heart disease, life attributable risk of cancer was 6.5% above baseline [4]. In pediatric cardiology, cardiac catheterizations contribute to 60% of the total radiation exposure. Thus, patients included in this study are exposed to higher risks of cancer development compared to the general population. Therefore, the dose reduction of the 3DRA solely with 1 mSv and almost 10 mSv for the entire procedure is highly relevant.

### 3DRA and Total Catheterization EDs

Low 3DRA EDs can be achieved using simple 2D angiography dose-lowering techniques and can, therefore, be easily applied in every cathlab. EDs of 3DRA have not been extensively calculated and reported in pediatric cardiology. Compared to other studies, our 3DRA ED is low. Table 3 provides an overview difference 3DRA ED values reported by previous studies. Reinke et al. suggested that an ED lower than 1 mSv is possible for 3DRA in real patients [18]. Our study is first to confirm this hypothesis and to show ED can be decreased to a minimum using simple techniques without any diagnostic image quality loss.

**Table 3:** Comparison of studies on ED in 3DRA

Author	Year of publication	Patients	Number of patients	Measure of center	ED (mSv)
Eloot [9]	2013	Adults	40	Median	5.7
Wielandts [13]	2010	Adults	42	Mean	6.6
De Buck [19]	2013	Adults	40 (75% left atrium)	N/A	2.6 (left atrium group) 1.2 (right atrium group)
Haddad [10]	2016	Pediatric patient; age unknown	1	-	1.8
Surendran [20]	2017	≤ 2 years	15	Median	1.35

*N/A* not available

Watson et al. compared computed tomographic angiography (CTA) with a diagnostic cardiac catheterization in pediatric patients. A median calculated ED of 0.74 mSv and 10.8 mSv was found for CTA and catheterization, respectively [21]. Ait-Ali et al. reported a total estimated ED for a diagnostic catheterization of 4.6 mSv in pediatric patients [22]. Our median total ED was 0.86 mSv in our diagnostic group and is comparable to the ED of CTA and much lower than the EDs of diagnostic catheterizations previously reported. Furthermore, in our study angiography ED was also significantly decreased. Thus, it seems that the excellent 3DRA image quality of this low-dose protocol reduces the need for additional angiographic imaging and the radiation burden of diagnostic catheterizations is comparable to CTA.

### Image Quality

Even though 90% of the scored items received the same score, interobserver variability was fair. The small number of rating categories is the most reasonable explanation. Interestingly, image quality was better in the low-dose cohort. Enhanced operator experience and optimization of the post-processing process could explain this. Moreover, better protocols were available leading to optimal diagnostic image quality [23].

### Correlations with ED

3DRA ED could be estimated with simple values as DAP and weight, which are readily available in every cathlab. Previously, Wielandts et al. found no relationship in adult patients between DAP and ED [13]. However, Peters et al. in children and Eloot et al. in adults found comparable Pearson’s correlation coefficients (*r*) of 0.87 and 0.92, respectively [9, 14]. Recently, Haddad et al. reported Pearson’s correlation coefficients ranging from 0.67 (aged > 15 years) to 0.98 (aged < 5 years) in 100 phantoms [10]. This confirms our finding that DAP seems to predict the ED best in the youngest patients. 3DRA is a standardized procedure and therefore DAP is likely to correlate well to ED. Correlations between ED and skin dose have not been widely published. Only from Peters et al. study a Pearson’s correlation coefficient of 0.91 can be derived, which is slightly higher than our Pearson’s correlation coefficient of 0.85.

### Strengths and Limitations

One of the strengths of this study is the large group of patients. This is the first study reporting 3DRA EDs from 100 patients. A fair comparison could be made with previously reported 3DRA EDs from the same center where patients underwent the same 3DRA protocol except for the above-mentioned factors. Calculation of ED was done according to the current standard [3]. A limitation of this study is the heterogenous patient group and, therefore, patients could not be directly matched to patients from the control cohort. However, the anatomical roadmap required for the procedures does not differ enormously, nor can the 3DRA procedure. This would not affect 3DRA ED values greatly. Second, ED calculations have been done based on phantom models corrected for height and weight, but not for the exact age and gender. Third, our study was based on certain protocols and equipment, which not every institution might use. This makes comparison and reproducibility harder.

## Conclusions

Standardized use of traditional 2D angiography dose-lowering techniques leads to a 66% dose reduction in 3DRA with preserved image quality and a significant decrease in total procedural radiation dose. DAP and skin dose are reliable predictors of ED. The usage of the dose-reducing steps described in this study is strongly advised.

## Electronic supplementary material

Below is the link to the electronic supplementary material.

Supplementary material 1 (DOCX 16 KB)

## Abbreviations

3DRA: Three-dimensional rotational angiography
DAP: Dose area product
ED: Effective dose
