# Supplementary material for: Dramatic Dose Reduction in Three-Dimensional Rotational Angiography After Implementation of a Simple Dose Reduction Protocol
Source: Pediatr Cardiol. 2018 Aug 3;39(8):1635–41. doi: 10.1007/s00246-018-1943-3 (PMC6244991; doi:10.1007/s00246-018-1943-3)
Supplement: Supplementary file 1 — Supplementary material 1 (DOCX 16 KB) [file 246_2018_1943_MOESM1_ESM.docx]

**Dramatic Dose Reduction in Three-Dimensional Rotational Angiography After Implementation of a Simple Dose Reduction Protocol.**

**Pediatric Cardiology**

Savine C.S. Minderhoud^1^, MD; Femke van der Stelt^1^, MD; Mirella M.C. Molenschot^1^, MD; Michel S. Koster^2^; Gregor K. Krings^1^, MD, PhD; Johannes M.P.J. Breur^1^, MD, PhD

**Author Affiliations:**

From the ^1^Department of Pediatric Cardiology, Wilhelmina Children’s Hospital, University Medical Center, Utrecht, The Netherlands; and ^2^Radiation protection and consultancy, NRG-Consultancy and Services, Petten, The Netherlands.

**Email address corresponding author: savineminderhoud@gmail.com**

**Supplemental material**

| Table S1. Weight protocols | |
| --- | --- |
| Weight protocol (kg) | **Tube voltage (kV)** |
| <10 | 70 |
| <20 | 70-77 |
| <30 | 81-96 |
| >30 | 91-93 |
